# Identification of Key Genes of Fruit Shape Variation in Jujube with Integrating Elliptic Fourier Descriptors and Transcriptome

**DOI:** 10.3390/plants13091273

**Published:** 2024-05-05

**Authors:** Yue Ren, Wenqing Fu, Yi Gao, Yuhan Chen, Decang Kong, Ming Cao, Xiaoming Pang, Wenhao Bo

**Affiliations:** 1 State Key Laboratory of Tree Genetics and Breeding, National Engineering Research Center of Tree Breeding and Ecological Restoration, Key Laboratory of Genetics and Breeding in Forest Trees and Ornamental Plants, Ministry of Education, College of Biological Sciences and Biotechnology, Beijing Forestry University, Beijing 100083, China; yueren9918@bjfu.edu.cn (Y.R.); wenqingfu@bjfu.edu.cn (W.F.); gy18335481527@163.com (Y.G.); chenyuhan2023@bjfu.edu.cn (Y.C.); xmpang@bjfu.edu.cn (X.P.); 2 National Foundation for Improved Cultivar of Chinese Jujube, Cangzhou 061000, China; kongdecang@126.com (D.K.); i2008caoming@126.com (M.C.)

**Keywords:** EFDs, WGCNA, STEM, fruit shape

## Abstract

Jujube (*Ziziphus jujuba*) exhibits a rich diversity in fruit shape, with natural occurrences of gourd-like, flattened, and other special shapes. Despite the ongoing research into fruit shape, studies integrating elliptical Fourier descriptors (EFDs) with both Short Time-series Expression Miner (STEM) and weighted gene co-expression network analysis (WGCNA) for gene discovery remain scarce. In this study, six cultivars of jujube fruits with distinct shapes were selected, and samples were collected from the fruit set period to the white mature stage across five time points for shape analysis and transcriptome studies. By combining EFDs with WGCNA and STEM, the study aimed to identify the critical periods and key genes involved in the formation of jujube fruit shape. The findings indicated that the D25 (25 days after flowering) is crucial for the development of jujube fruit shape. Moreover, *ZjAGL80*, *ZjABI3,* and eight other genes have been implicated to regulate the shape development of jujubes at different periods of fruit development, through seed development and fruit development pathway. In this research, EFDs were employed to precisely delineate the shape of jujube fruits. This approach, in conjunction with transcriptome, enhanced the precision of gene identification, and offered an innovative methodology for fruit shape analysis. This integration facilitates the advancement of research into the morphological characteristics of plant fruits, underpinning the development of a refined framework for the genetic underpinnings of fruit shape variation.

## 1. Introduction

Fruit shape is a fundamental characteristic of plants, with significant implications for genetic research and the identification of regulatory genes associated with morphogenesis. Investigating the critical biological processes underlying fruit shape formation is essential for species morphogenesis during growth and development and for the selection and breeding of novel cultivars. Fruit shape is an important trait for consumers and attracts considerable attention from geneticists and breeders [1,2]. Research indicates that fruit shape results from the interaction of genetic and environmental factors, contributing to the diverse fruit morphologies observed in nature [3]. Fruit development initiates with cell differentiation in floral organs, continuing from flowering through to fruit maturity and senescence [4,5]. The dynamics of cell division, cell size, and cell number influence fruit shape during growth [6]. Genetic studies of fruit shape across various horticultural crops have identified numerous genes regulating fruit development [7,8]. In tomatoes (*Solanum lycopersicum*), the *SUN* gene impacts cell distribution along the proximal–distal axis, while the *LOCULE NUMBER* (*LC*) and *FASCIATED* (*FAS*) genes modulate fruit shape through ovary number control [9,10,11].

The jujube (*Ziziphus jujuba*) is an economically significant species, exhibiting a wide array of fruit shapes, from spherical and elliptical to gourd-shaped, flat, and inverted heart shapes. However, the abundance of fruit shape variation also poses significant difficulties in the study of jujube fruit shape. Currently, there is still a lack of classification criteria for jujube fruit shape. The progression of a drupe’s growth and maturation, spanning from the period of flower opening to full ripeness, is commonly depicted as consisting of two exponential phases within a double sigmoid curve in jujube. The first stage begins with the flowering of the jujube, encompassing rapid growth of the jujube fruit; the second stage involves relatively slow fruit growth, primarily characterized by cell elongation and thickening of the cell wall; the third stage entails continuous cell expansion within the fruit, leading to the formation of the final fruit structure [12]. In blueberries with the same double ‘S’ developmental pattern, we found that there were different changes in fruit shape between the two growth spurts, and the growth of fruit transverse and longitudinal diameters was basically the same as that of the double ‘S’, but with a decreasing fruit shape index. This also suggests that there are different trends in fruit shape at different stages of growth and development [13]. The size of jujube fruit, as a fundamental characteristic, has garnered widespread attention, leading to the discovery of multiple genes and QTLs associated with fruit size regulation [14]. However, little is known about the genes that influence the shape of jujube fruit currently. Therefore, exploring genes related to jujube fruit shape is of significant importance for understanding the mechanism of fruit shape formation in plants and for the breeding of new jujube cultivars.

In addition to internal genetic factors, plant fruit shape is also influenced by external factors. Fruit shape development is intricately linked to environmental conditions, nutrition, and hormonal influences [15]. It has been demonstrated that the rational application of exogenous hormones can have an effect on the shape of plant fruits [16]. Plant hormones, including gibberellins, cytokinins, and auxins, are continuously produced in the seed, stimulating tissue growth and development around the fruit [17]. Fluctuating endogenous hormone levels in the fruit during different growth stages impact fruit shape. The role of hormones in fruit morphology varies, with gibberellins promoting cell elongation and fruit lengthening [16,18]. These phytohormones act both individually and synergistically to regulate plant fruit growth and development.

Fruit shape, as a complex trait, often defies straightforward description, with traditional methods relying on descriptive adjectives [19,20]. However, simplistic geometric shapes fall short of capturing the comprehensive form of fruits, and such rudimentary descriptions are highly subjective, posing significant challenges to the genetic study of fruit shape. To advance the understanding of fruit morphology, the concept of fruit shape index (FSI) was progressively incorporated into descriptions, initially applied in studies of capsicum shape and subsequently employed in research on melons, tomatoes, apples, and other crops [21,22,23]. Nevertheless, the FSI, reflecting characteristics at specific loci, fails to convey the complete three-dimensional shape of fruits and requires extensive experience from agronomists to define any given species.

To fulfill the need for a more precise and universally applicable description of fruit shape, we adopted the mathematical approach of elliptical Fourier descriptors (EFDs), transforming the vague shape information into precise numerical data by representing fruit shapes as closed contours and analyzing them with elliptical Fourier coefficients [24]. Fourier descriptors encode the shape of two-dimensional objects through the Fourier transformation of their boundaries, decomposing a curve into a sum of ellipses associated with harmonics, each producing four coefficients for multivariate statistical analysis [25]. This method enables detailed analysis and description of closed contours through superimposed elliptical Fourier coefficients. Fourier descriptors are widely used for shape analysis, offering high accuracy and precision due to their sensitivity to orientation and scale [26]. EFDs have been applied across various species, including strawberries and grapes, accelerating the pace of research in fruit shape [27]. However, integrating EFDs with transcriptomic data for the exploration of regulatory genes in plant fruit shape remains infrequent, particularly in jujube research.

In this study, we selected six jujube cultivars representing diverse fruit shapes, collected plant materials throughout fruit development, and performed transcriptomic analysis. By integrating the mathematical model of EFDs, we analyzed the shapes of jujube fruits across different developmental stages. We constructed co-expression networks for genes associated with fruit development at various stages and shapes, identifying key genes related to jujube fruit morphology. Additionally, we analyzed gene expression trends in jujube fruits of different shapes at the same developmental stage, comparing gene expression variations. By combining weighted gene co-expression network analysis (WGCNA) and Short Time-series Expression Miner (STEM), we identified *ZjAGL80*, *ZjABI3*, and the other eight candidate genes associated with the jujube fruit shape. This research not only holds significant scientific and practical value for the genetic development of jujube fruits but also offers insights for breeding programs targeting fruit shapes in economically important fruit trees in China.

## 2. Results

### 2.1. Image Processing and Extraction of EFDs

**Figure 1:**
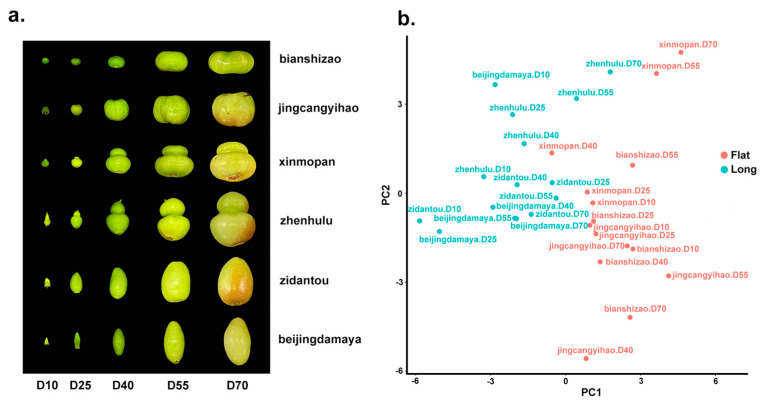
(**a**) Representatives of D10-D70 developmental periods of six different cultivars of jujube fruit; D represents the number of days since flowering. (**b**) PCA plot of fruit shape resolved EFDs for six different cultivars of jujube fruit at D10-D70 periods.

All collected plant materials were photographed under identical conditions, yielding uniformly formatted images (Figure 1a). Using Python 3.10, the images were processed to extract the contours of jujube fruits. Following orientation and area corrections, all fruit samples were standardized to a contour internal area of 70,000 pixels to minimize errors in the elliptical Fourier coefficient analysis due to size variations. Furthermore, orientation corrections were applied to all contours, aligning them vertically based on symmetry to eliminate the sensitivity of the elliptical Fourier coefficients to the direction of the contour, thereby enhancing analytical accuracy. The corrected contours were uniform in area and orientation. An average contour was computed for three fruits of each variety, and the elliptical Fourier coefficients were calculated for these average contours, extracting levels 1 to 4 of elliptical Fourier coefficients, each level yielding four Fourier parameters, a, b, c, and d, resulting in a total of 16 sets of Fourier parameters.

Principal component analysis (PCA) was conducted on the 16 sets of elliptical Fourier coefficients to evaluate shape differences during the developmental process of jujube fruits among different cultivars. Based on the PCA results, the six jujube cultivars were categorized into groups according to the PC1–PC2 plot (Figure 1b). The fruit length-to-width ratios were measured from the original photographs, revealing that ‘xinmopan’, ‘jingcangyihao’, and ‘bianshizao’ had ratios less than 1 at all stages, while ‘zidantou’, ‘beijingdama ya’, and ‘zhenhulu’ had ratios greater than 1, consistent with the PCA outcomes. Thus, 30 samples were distinctly divided into two groups: ‘xinmopan’, ‘jingcangyihao’, and ‘bianshizao’ formed a group characterized by lateral development (flat group), while ‘zidantou’, ‘beijingdamaya’, and ‘zhenhulu’ formed another group indicative of vertical development (long group). In addition, we counted the FSI of six different shapes of jujube fruits at different developmental periods and found that the shapes of the fruits kept changing and the FSI varied during different developmental periods (Figure S1).

### 2.2. WGCNA Analyses during the Whole Developmental Period of Jujube Fruits of Different Shapes

**Figure 2:**
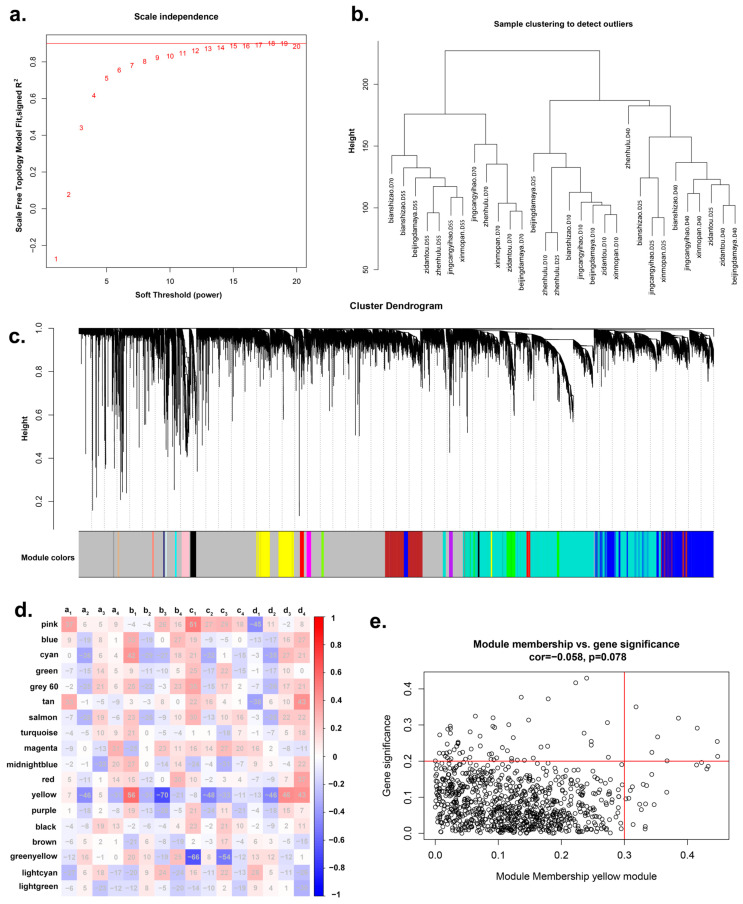
(**a**) Analyses of network topology for various soft-thresholding powers (weighting coefficient (β), with the scale-free topology criterion roughly set at 0.9. 18 being the best-fitting power. (**b**) Sample clustering plot of 30 jujube samples. (**c**) Cluster dendrogram and module assignment. (**d**) Correlation row heatmap between 18 modules and 16 sets of EFDs parameters. (**e**) GS–MM scatter plot of yellow module; genes with GS > 0.2 and MM > 0.3 were selected as candidate genes.

Transcriptomic sequencing was conducted on jujube fruits from five developmental stages, representing six distinct jujube cultivars. Each sample consisted of a mixture of three fruits. Subsequently, the expression levels of all genes were quantified. Observation revealed significant expression differences in some genes across various shapes and developmental stages. After calculation, 18 was selected as the soft threshold for WGCNA analysis (Figure 2a). Hierarchical clustering analysis confirmed that the gene expression patterns of all 30 samples were as expected, with no outliers detected; the clustering generally corresponded to the period in which the material was collected and could therefore be used for further analyses (Figure 2b). The clustering of jujube fruit samples with different shapes at the same developmental stage confirmed that all plant materials conform to the growth and development period. And the jujube fruits at the same developmental stage exhibited similar patterns of gene expression. Weighted gene co-expression network analysis (WGCNA) was performed to cluster genes with similar expression patterns, identifying 18 gene clusters (Figure 2c).

**Figure 3:**
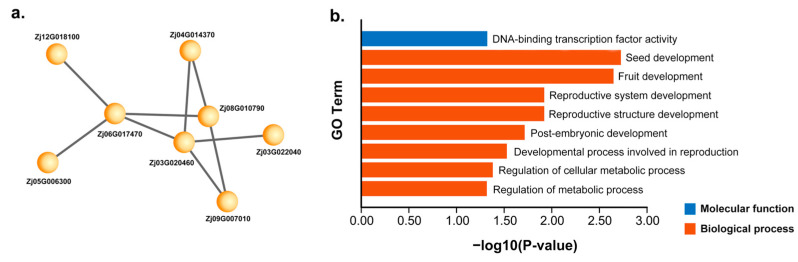
(**a**) Network depiction of the pink module. (**b**) Bar graph of GO enrichment analysis of 79 candidate genes.

Correlation analysis between all gene clusters and the 16 sets of elliptical Fourier coefficients revealed that three clusters exhibited strong associations with the EFDs (Figure 2d). Fourier coefficient b3 showed the highest correlation with the yellow module (−70%). Fourier coefficient c1 was notably correlated with the pink module (51%) and the greenyellow module (−66%). Fourier coefficient c3 had a −54% correlation with the greenyellow module. Fourier coefficient b1 exhibited a significant correlation with the yellow module (56%). Consequently, the yellow, pink, and greenyellow modules were selected as candidate modules for further analysis and mapped the corresponding gene networks (Figure 3a). *ZjHSD5* (Zj06G017470) was identified as the hub gene of the pink cluster. Based on protein sequence alignment, this gene is a homologue of *AtHSD5* in Arabidopsis (*Arabidopsis thaliana*), which has been shown to regulate plant growth and development and to regulate ABA and increase plant sensitivity to BRs [28]. ABA can achieve regulation of plant fruit shape by regulating cell expansion and other pathways [29].

Further selection within the three candidate clusters was carried out by calculating each gene’s gene significance (GS) within the module, and the absolute correlation of phenotype with module membership (MM), followed by plotting a GS–MM scatter plot. Using a threshold of GS > 0.2 and MM > 0.3, 79 candidate genes associated with jujube fruit shape were identified from the GS–MM scatter plot (Figure 2e).

Gene ontology (GO) enrichment analysis of the 79 candidate genes revealed their concentration in several metabolic pathways, including DNA-binding transcription factor activity, seed development, fruit development, reproductive system development, reproductive structure development, post-embryonic development, developmental processes involved in reproduction, regulation of the cellular metabolic process, and regulation of the metabolic process. Notably, the most significant enrichment terms were seed development and fruit development (Figure 3b).

### 2.3. STEM Analysis during the Whole Developmental Period of Jujube Fruits of Different Shapes

**Figure 4:**
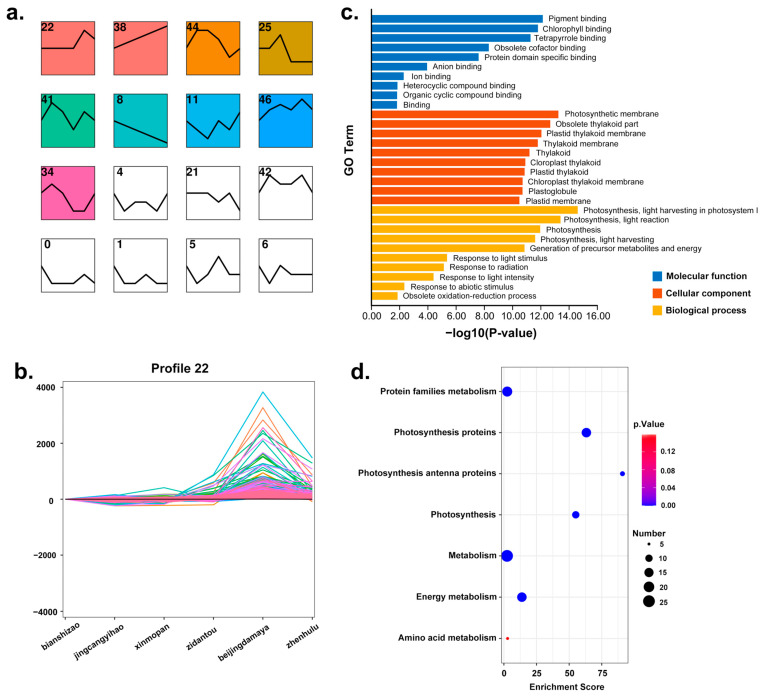
(**a**) Trend analysis of 27,174 genes of six jujube fruit cultivars (‘bianshizao’, ‘jingcangyihao’, ‘xinmopan’, ‘zidantou’, beijingdamaya’, and ‘zhenhulu’) at the D25 developmental stage. Each square represents a gene profile, the number within the square represents the cluster number, and the folded line in the square is the gene expression trend line in the cluster. The images show the 16 profiles, and the full profile diagram is in the accompanying Figure S2. (**b**) Gene expression trend in profile22. The image is an enlargement of the first profile in Figure 4a. The horizontal coordinates represent each of the six different jujube cultivars (‘bianshizao’, ‘jingcangyihao’, ‘xinmopan’, ‘zidantou’, beijingdamaya’, and ‘zhenhulu’), and the vertical coordinates are the gene expression levels during the D25 developmental stage and after normalization. (**c**) Bar graph of GO enrichment analysis of 118 candidate genes. (**d**) KEGG enrichment analysis of 118 candidate genes.

For the STEM analysis, to assess gene expression variability across different jujube fruit shapes during development, 30 jujube fruit samples were analyzed for gene expression trends. At each of the five developmental stages, gene expression trends were analyzed across six distinct shapes of jujube fruit. Throughout the entire growth and development period, from the fruit set to the white mature stage, varying gene expression patterns were observed at different stages. In STEM analysis, 27,174 genes from the jujube genome were clustered into various modules based on expression pattern differences, with significant enrichment observed in some clusters (Figure 4a and Figure S2–S6).

PCA analysis of the elliptical Fourier descriptors (EFDs) for the jujube fruit samples from different cultivars and stages distinguished two groups: ‘xinmopan’, ‘jingcangyihao’, and ‘bianshizao’ formed the flattened fruit group, while ‘zidantou’, beijingdamaya’, and ‘zhenhulu’ constituted the elongated fruit group (Figure 1b). Candidate gene screening between the two groups identified candidate genes at different time points: 9 genes at D10, 71 genes at D25, 16 genes at D40, 13 genes at D55, and 4 genes at D70, totaling 118 candidate genes across five developmental stages.

Gene function enrichment analysis (GO) and metabolic pathway enrichment analysis (KEGG) were conducted for 118 candidate genes in all candidate modules (Figure 4c,d). Fifty-four GO terms were enriched, with the most significant in the cellular component category being photosynthetic membrane, obsolete thylakoid part, and plastid thylakoid membrane. In the biological process category, genes were significantly enriched in photosynthesis and response to light stimulus, while in the molecular function category, the most enriched terms were pigment binding, chlorophyll binding, and tetrapyrrole binding. These GO terms are primarily related to plant photosynthesis, influencing plant photomorphogenesis and, consequently, jujube fruit shape variation. KEGG enrichment analysis revealed significant concentration in seven metabolic pathways: photosynthesis proteins, photosynthesis, energy metabolism, photosynthesis antenna proteins, metabolism, protein families: metabolism, and amino acid metabolism, aligning with the GO enrichment findings and highlighting the focus on plant photomorphogenesis.

## 3. Discussion

### 3.1. EFDs Can More Accurately Describe Fruit Shapes

In this study, EFDs were utilized to extract and analyze the contours of jujube fruits from six different cultivars at five developmental stages. Compared to traditional descriptive methods, such as the FSI, EFDs offer a more accurate analysis of shape, representing a lossless information method. With the increasing order of Fourier coefficients, the accuracy of contour analysis likewise improves. PCA of the elliptical Fourier coefficient analysis results for plant samples accurately divided the samples into two groups, consistent with the fruit shape development trends observed during the growth of jujube fruits. The PCA results for the elliptical Fourier coefficients PC1 and PC2 were highly correlated with the fruit’s aspect ratio, clustering 15 samples from ‘xinmopan’, ‘jingcangyihao’, and ‘bianshizao’ over five stages into one group. Observations from sampling images showed that these three cultivars had a significantly greater horizontal than vertical diameter upon maturity, presenting a flattened shape, with lateral growth predominating throughout the development of the jujube fruit. Conversely, 15 samples from ‘zidantou’, ‘beijingdamaya’, and ‘zhenhulu’ over five stages were classified into another group, where these cultivars exhibited a greater vertical than horizontal diameter upon maturity, indicating an elongated shape, with vertical growth more pronounced from fruit set through development. The PCA results for EFDs, combined with the growth patterns of the fruit, allow for the division of the 30 samples into two groups. Additionally, statistical analysis of the EFDs confirmed that the distribution of all parameters for the 30 samples conformed to a normal distribution, as expected. These findings validate the accuracy of EFDs for contour description and their feasibility for analyzing and researching plant fruit shapes.

WGCNA was performed on the transcriptome data of 30 samples, clustering 27,174 genes into 18 modules based on gene expression patterns. The correlation between each module and the EFDs of the jujube fruit contours was calculated. The analysis revealed that three modules showed a high correlation with the EFDs. Specifically, the Fourier coefficient b3 had the highest correlation with the yellow module at −70%, while the coefficient c1 had a −66% correlation with the greenyellow module and a 51% correlation with the pink module. Additionally, Fourier coefficient b1 exhibited a 56% correlation with the yellow module, and coefficient c3 had a −54% correlation with the greenyellow module. These results indicate the feasibility of using EFDs for plant fruit contour analysis and suggest a strong likelihood that the yellow, pink, and greenyellow modules are related to the shape of jujube fruits.

### 3.2. WGCNA Analysis of Jujube Fruits of Different Periods and Shapes to Obtain Key Metabolic Pathways Related to Jujube Fruit Shape

Based on the GS–MM scatter plot, 79 candidate genes related to jujube fruit shape were identified. GO enrichment analysis of these hub genes showed enrichment in nine terms, with a significant concentration in the molecular function category for DNA-binding transcription factor activity and in biological processes for seed development, fruit development, reproductive system development, and reproductive structure development (Figure 3b). Notably, seed development (GO:0048316) was enriched as a subset of fruit development (GO:0010154). In Figure 3a, the gene *ZjHSD5* (Zj06G017470) was mined as a hub gene of the pink cluster, and based on gene sequence comparison, *ZjHSD5* is homologous to *AtHSD5* in Arabidopsis. *AtHSD5* has been shown to regulate plant growth and development and to regulate ABA and increase plant sensitivity to BRs [28]. *ZjTZF6* (Zj03G020460) is a homologous gene of the Arabidopsis *AtTZF6* gene and a member of the CCCH-type zinc finger gene family. Previous studies have demonstrated the involvement of this gene family member in methyl jasmonate treatments [30]. In Arabidopsis, *AtTZF6* functions as a positive regulator of ABA and a negative regulator of seed germination response mediated by light and GA [31]. GA has been found to have an effect on fruit shape by adjusting fruit cell size and cell number [32].

Research indicates that various plant hormones, including gibberellins, cytokinins, and auxins, are produced in plant seeds, stimulating the growth and development of surrounding tissues. By regulating the distribution and concentration of plant hormones within fruit tissues, plants can achieve shape variation [17]. The gene *ZjLEC2* (Zj01G016210), enriched in seed development (GO:0048316), was identified as a B3 domain-containing transcription factor through protein sequence alignment. B3 domain-containing transcription factors, first discovered in the maize Vp1 (Viviparous-1) gene, are crucial in seed growth and development [33]. In addition, the gene *ZjABI3* (Zj09G007010) has been found to be the ABI3 gene. The ABI3 member of the B3 domain-containing transcription factor family, an ABA response factor, significantly regulates seed development, thereby influencing fruit shape [34].Seed development plays a vital role in fruit development and significantly impacts the variation in plant fruit shape [35]. These findings suggest that these genes could play a regulatory role in the development of jujube fruit shape. Moreover, this study demonstrates the high accuracy and feasibility of using EFDs for fruit shape analysis, contributing to the advancement of research on plant fruit shape.

### 3.3. STEM Analysis for Mining Candidate Genes of Jujube Fruit Shape Variation

STEM analysis revealed significant differences in gene expression among jujube fruits of different shapes at the same developmental stages, with distinct gene expression patterns observed throughout the growth period from the fruit set to the white mature stage. PCA of the fruit shape using EFDs categorized six jujube cultivars into two groups, “flat” and “long” (Figure 1b). This study conducted a horizontal comparison of gene expression patterns for six jujube fruits with shape differences within the same period, facilitating the identification of genes that regulate the development of different jujube fruit shapes.

Candidate gene expression analysis between the D10 and D70 stages yielded 118 candidate genes, showing significant expression differences between the two sample groups. Of these, 9 genes were identified at D10, 71 genes at D25, 16 genes at D40, 13 genes at D55, and 4 genes at D70. The D25 stage had the highest number of candidate genes, and the differences in gene expression trends during the D25 period were very pronounced (Figure 4a,b), suggesting it may be a critical period for the development of jujube fruit shape. The STEM clustering at D25 (Figure 3a) divided the genes into 50 profiles, with 15 significant profiles. Some profiles exhibited a gene expression pattern consistent with the shape grouping; for example, in profile22, ‘xinmopan’, ‘jingcangyihao’, and ‘bianshizao’ showed consistently lower gene expression, while ‘zidantou’, ‘beijingdamaya’, and ‘zhen hulu’ exhibited higher expression levels (Figure 3b). This provides strong evidence for candidate gene selection and further supports the hypothesis that D25 is a key period for the development of jujube fruit shape.

### 3.4. The Genes Affecting the Shape of Jujube Fruit Were Discovered

Combining WGCNA and STEM analyses for further selection identified 10 candidate genes possibly related to jujube fruit shape. Protein sequence alignment revealed that gene *ZjTZF6* (Zj03G020460) encodes a zinc finger CCCH domain-containing protein and may be involved in regulating cell signaling pathways, affecting growth, differentiation, and apoptosis. In poplar, C3H14 and C3H15 have been associated with anther development, while *AtC3H14* in Arabidopsis relates to cell elongation [36]. Zj01G007630 encodes a plastid lipid-associated protein, with the FBN1 gene in bell peppers identified as promoting fruit ripening [37]. The gene *ZjAGL80*(Zj06G022170) and gene *ZjAGL104*(Zj06G004120) encode the agamous-like MADS-box protein. *ZjAGL80* is a homolog of Arabidopsis thaliana *AtAGL80*. The MADS-box gene family has been proven to be crucial in plant fruit development, influencing both fruit ripening and early developmental stages [38]. *ZjHHO5* (Zj04G014370) is a homologous gene of *HRS1* and participates in ABA signal transduction in plants [39]. *ZjATL12* (Zj06G009700) is a homologous gene of the Arabidopsis gene *ATL12*, and studies have shown that *ATL12* is involved in the ABA signaling pathway in Arabidopsis [40]. Gene *ZjAHG1* (Zj03G022040) has been identified as a member of the *AHG* family and serves as a central negative regulatory factor in the ABA response of plant seeds [41]. In addition, the genes *ZjHSD5*, *ZjLEC2*, and *ZjABI3* have also been implied to regulate the growth and development of plant fruits.

In addition, a total of 10 genes, including *ZjHSD5*, *ZjLEC2*, *ZjABI3,* have been found to be associated with fruit shape development. These findings support the hypothesis that the identified candidate genes are key regulators of jujube fruit shape.

## 4. Materials and Methods

### 4.1. Jujube Sample Collection and Library Preparation

In this study, fruits of six jujube cultivars with distinct fruit shape characteristics were collected at the National Jujube Breeding Base in Cangzhou, Hebei, China. Fruits of the selected varieties, namely ‘xinmopan’, ‘jingcangyihao’, ‘bianshizao’, ‘zidantou’, ‘beijingdamaya’, and ‘zhenhulu’, were selected for transcriptome analysis. Fruit samples were collected over five developmental stages, from fruit set to white mature stage, denoted as D10 (10 days after flowering), D25 (25 days after flowering), D40 (40 days after flowering), D55 (55 days after flowering), and D70 (70 days after flowering). Each sample consisted of a mixture of three healthy, disease-free, and insect-free fruits collected from jujube trees at each developmental stage. The collected fruits were photographed under identical conditions and rapidly frozen with liquid nitrogen, then stored at −80 °C.

### 4.2. Jujube EFDs Analysis

Following collection, the plant materials were immediately photographed using the same equipment. For EFD analysis, three fruits were selected as representative samples for each developmental stage per variety. For image distortion correction, a 6 cm × 6 cm standard checkerboard was used as a reference. The corrected images were segmented to isolate individual jujube fruits. Using the Otsu method [42], the segmented images were binarized to convert the jujube fruits into closed contours. Given the sensitivity of EFDs to the size and orientation of contours, it was necessary to normalize these factors: the pixel count within all closed contours was standardized to 70,000, and the orientation of the contours was aligned vertically based on symmetry. The contours of three fruits per sample were computed to obtain the average contour for that sample, which was then used to calculate the elliptical Fourier coefficients. Four levels of elliptical Fourier coefficients were extracted for each average contour, with each level comprising four parameters. All these procedures were conducted in a Python 3.10 (Python Release Python 3.10.0|Python.org) environment.

### 4.3. PCA Analysis of Jujube EFDs

In this study, the EFDs of jujube fruit photographs from 6 different cultivars and 5 key developmental stages were analyzed, yielding 4 levels of EFDs for each, resulting in 16 sets of parameters for 30 samples. Statistical analysis and PCA clustering of these EFDs separated the 30 samples into 2 groups. The PCA results were visualized using the ggplot2 package [43] in R v4.3.0.

### 4.4. RNA Isolation and Transcriptome Analysis

Sequencing libraries for this study were prepared using the NEBNext^®^ Ultra™ RNA Library Prep Kit for Illumina^®^ (NEB, USA), followed by sequencing on the DNBSEQ-T7 platform. Quality control of the raw sequencing data was assessed using FastQC (https://www.bioinformatics.babraham.ac.uk/projects/fastqc/, accessed on 12 November 2022), with subsequent filtering performed by fastp [44]. The Jing39 jujube genome served as the reference for expression analysis. The genome index was created and sequencing data alignment was executed using HISAT2 [45]. Conversion of data formats was facilitated by SAMtools [46], while gene expression quantification was conducted with featureCounts. This process culminated in the determination of the FPKM expression value for each gene, providing a normalized measure of gene expression.

### 4.5. Weighted Gene Co-Expression Network Analysis

The WGCNA package [47] in R was used to construct a weighted gene co-expression network, analyzing the expression levels of all genes across the six cultivars and five key developmental stages. Clustering analysis based on FPKM values excluded outlier samples, and pairwise Pearson correlations were computed for all genes. With a scale-free topology fit index, 18 was selected as the soft threshold for further analysis. Genes were hierarchically clustered based on TOM similarity, and modules were detected using the dynamic tree cutting method (mergeCutHeight = 0.25, minModuleSize = 30). Modules with over 80% similarity were merged after calculating each module’s eigengene. The correlation between each module and the four levels of EFDs was calculated and visualized using the ggplot2 package. Modules with high trait association were selected as candidate modules, and for each, gene significance (GS) and module membership (MM) were calculated. GS–MM scatter plots were drawn with MM and GS values on the x and y axes, respectively, with a GS > 0.5 and MM > 0.9 threshold to identify candidate genes. All these analyses were performed using R v4.3.0. Candidate modules were visualized using Cytoscape software [48].

### 4.6. STEM Analysis

The STEM software [49] was used for gene expression trend analysis of jujube fruits of different shapes at the same developmental stage. Six cultivars were divided into two groups: ‘xinmopan’, ‘jingcangyihao’, and ‘bianshizao’ were assigned to “flat” group, ‘zidantou’, ‘beijingdamaya’, and ‘zhenhulu’ were assigned to the “long” group. Gene expression trend analysis was conducted for the six samples at the same stage, categorizing 27,174 genes into various modules based on expression patterns. Based on the results of PCA grouping, the sum of FPKM values for genes in the significant profiles within each group was calculated separately. Additionally, the WGCNA package [44] calculated the correlation between each module and the EFDs, visualizing it in a heatmap to identify modules highly associated with trait parameters, using R v4.3.0.

### 4.7. GO and KEGG Analysis of Key Genes

To further explore candidate genes, TBtools [50] was employed for Gene ontology (GO) annotation and Kyoto Encyclopedia of Genes and Genomes (KEGG) pathway enrichment analysis. GO enrichment was categorized into biological process (BP), molecular function (MF), and cellular component (CC). The enrichment results were visualized using R v4.3.0.

## 5. Conclusions

This study employed the mathematical model of EFDs to analyze the shapes of different jujube fruits, converting shape information into numerical data. Various jujube fruits of different shapes were selected, and plant materials from multiple key stages throughout their developmental period were collected for WGCNA. Within the 18 clusters obtained through WGCNA, the yellow, pink, and greenyellow modules were identified as key modules for the regulation of jujube fruit shape. Correlation analysis with EFDs led to the identification of 79 candidate genes within these key clusters. GO enrichment analysis of these candidate genes suggests that they are likely critical regulators of jujube fruit shape development.

Subsequent analyses across various stages compared multiple shaped jujube fruits, utilizing transcriptome time-series analysis to identify differentially expressed genes within the same developmental stage. Throughout the fruit set to maturity stages, 118 candidate genes were identified, with the most genes discovered during the D25 young fruit stage, suggesting this is a critical period for jujube fruit shape development. In the D25 period, genes such as *ZjABI3* and *ZjAGL80* were shown to be more differentially expressed in different jujube cultivars.

Combining WGCNA and STEM, this study ultimately identified 10 key candidate genes that have been implicated to regulate jujube fruit shape. Among them, *ZjATL12* (Zj06G009700) is a member of the ABI family and regulates ABA metabolism in plants, and *ZjAHG1* (Zj03G022040) is a member of the *AHG* family and participates in the ABA pathway in plants. Genes Zj01G016210 and *ZjABI3* (Zj09G007010) are both B3 domain-containing transcription factors, playing regulatory roles in plant seed growth and development [33]. Meanwhile, gene Zj01G007630 regulates plant growth, development, and maturation. *ZjAGL80* (Zj06G022170) and *ZjAGL104* (Zj06G004120) are members of the MADS-box gene family, functioning in the early stages of plant growth and development as well as during maturation [51]. These genes are likely to play roles in different stages of jujube tree growth and development, collectively regulating jujube fruit shape. This study not only supports previous research on jujube trees and lays the foundation for the breeding of new jujube cultivars but also provides new insights into the study of plant fruit shape.

## Data Availability

Resequencing and transcriptome data used in the research are available in the NCBI SRA database under accession numbers PRJNA979988. The Jing39 genome is available at the NCBI under the accession number PRJNA979943.

## Supplementary Materials

The following supporting information can be downloaded at: https://www.mdpi.com/article/10.3390/plants13091273/s1, Figure S1: Changes in FSI of jujube fruits of different shapes at different developmental periods. The horizontal coordinates are the five developmental stages and the vertical coordinates are the mean values of the FSI; Figure S2: Trend analysis of 27,174 genes of six jujube fruit varieties (‘bianshizao’, ‘jingcangyihao’, ‘xinmopan’, ‘zidantou’, beijingdamaya’, and ‘zhenhulu’) at the D10 developmental stage; Figure S3: Trend analysis of 27,174 genes of six jujube fruit varieties (‘bianshizao’, ‘jingcangyihao’, ‘xinmopan’, ‘zidantou’, beijingdamaya’, and ‘zhenhulu’) at the D25 developmental stage; Figure 4: Trend analysis of 27,174 genes of six jujube fruit varieties (‘bianshizao’, ‘jingcangyihao’, ‘xinmopan’, ‘zidantou’, beijingdamaya’, and ‘zhenhulu’) at the D40 developmental stage; Figure S5: Trend analysis of 27,174 genes of six jujube fruit varieties (‘bianshizao’, ‘jingcangyihao’, ‘xinmopan’, ‘zidantou’, beijingdamaya’, and ‘zhenhulu’) at the D55 developmental stage; Figure S6: Trend analysis of 27,174 genes of six jujube fruit varieties (‘bianshizao’, ‘jingcangyihao’, ‘xinmopan’, ‘zidantou’, beijingdamaya’, and ‘zhenhulu’) at the D70 developmental stage. Table S1: Expression FPKM values of 27,174 genes in six varieties (‘bianshizao’, ‘jingcangyihao’, ‘xinmopan’, ‘zidantou’, beijingdamaya’, and ‘zhenhulu’) and five developmental periods (D10,D25,D40,D55 and D70) Table.
