# Supplementary material for: Identification of Key Genes of Fruit Shape Variation in Jujube with Integrating Elliptic Fourier Descriptors and Transcriptome
Source: Plants (Basel). 2024 May 5;13(9):1273. doi: 10.3390/plants13091273 (PMC11085141; doi:10.3390/plants13091273)
Supplement: Supplementary file 1 [file plants-13-01273-s001.zip › plants-2989430-supplementary/Supplementary Materials_figure.pdf]

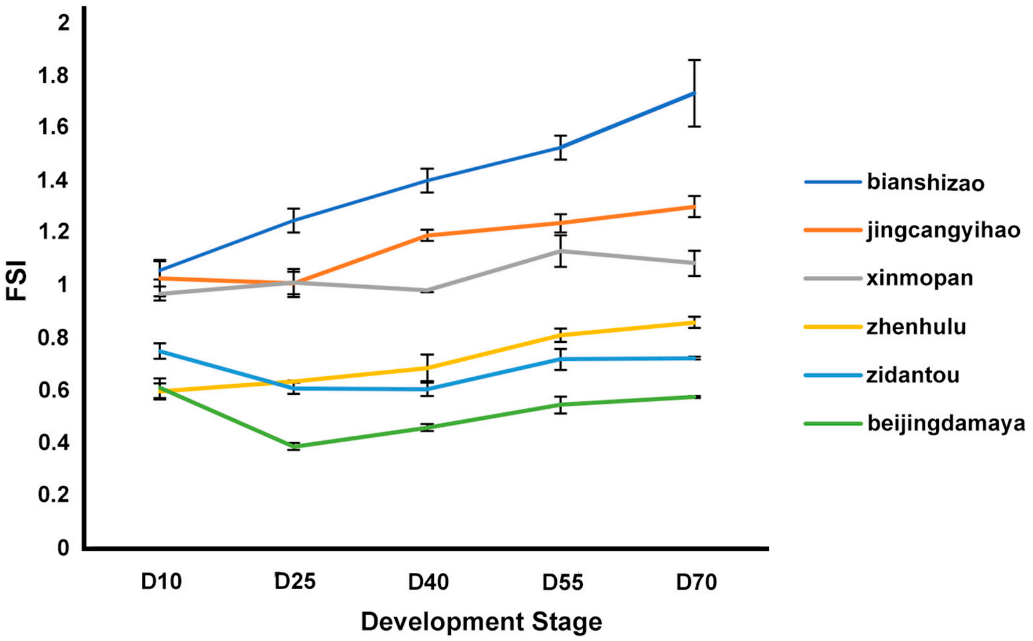

Figure S1. Changes in FSI of jujube fruits of different shapes at different developmental periods. The horizontal coordinates are the five developmental stages and the vertical coordinates are the mean values of the FSI.

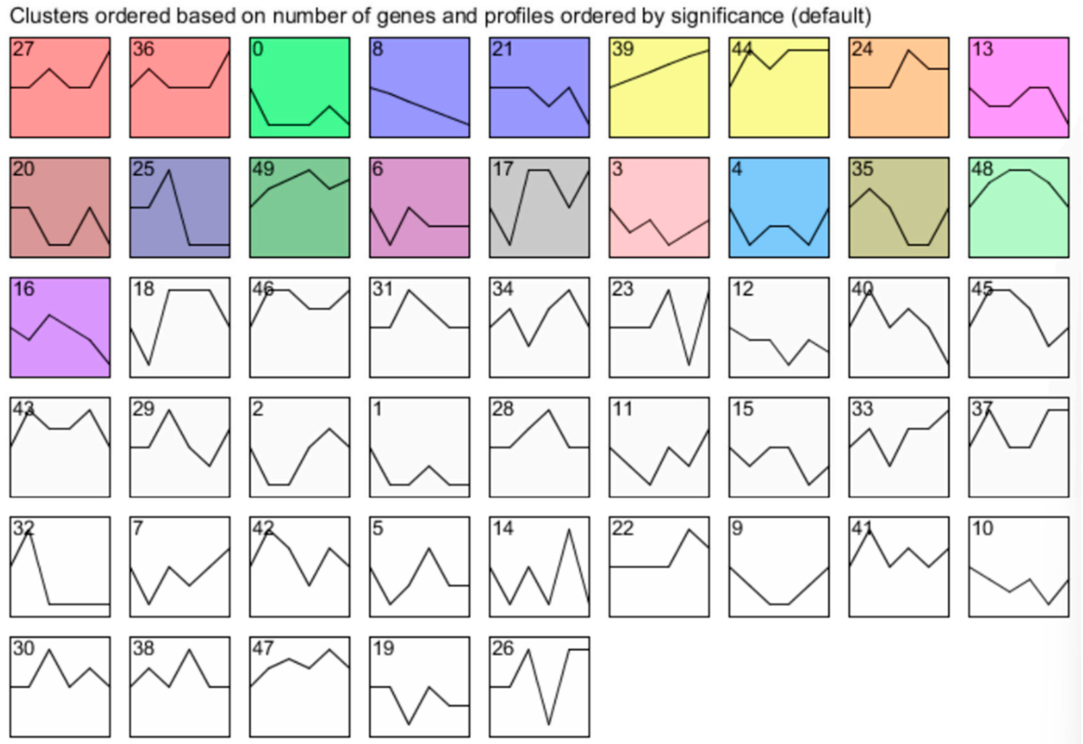

Figure S2. Trend analysis of 27174 genes of six jujube fruit varieties ('bianshizao', 'jingcangyihao', 'xinmopan', 'zidantou', beijingdamaya', and 'zhenhulu') at the D10 developmental stage.

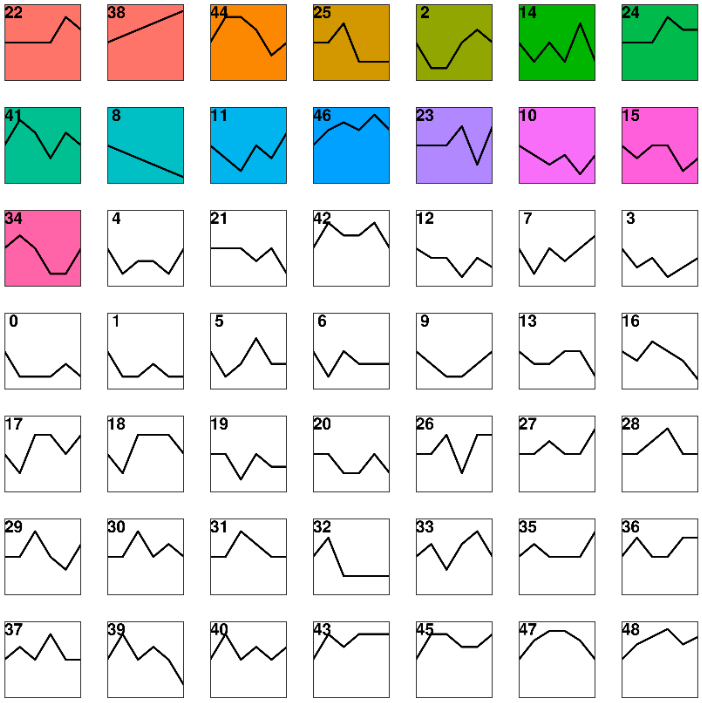

Figure S3. Trend analysis of 27174 genes of six jujube fruit varieties ('bianshizao', 'jingcangyihao', 'xinmopan', 'zidantou', beijingdamaya', and 'zhenhulu') at the D25 developmental stage.

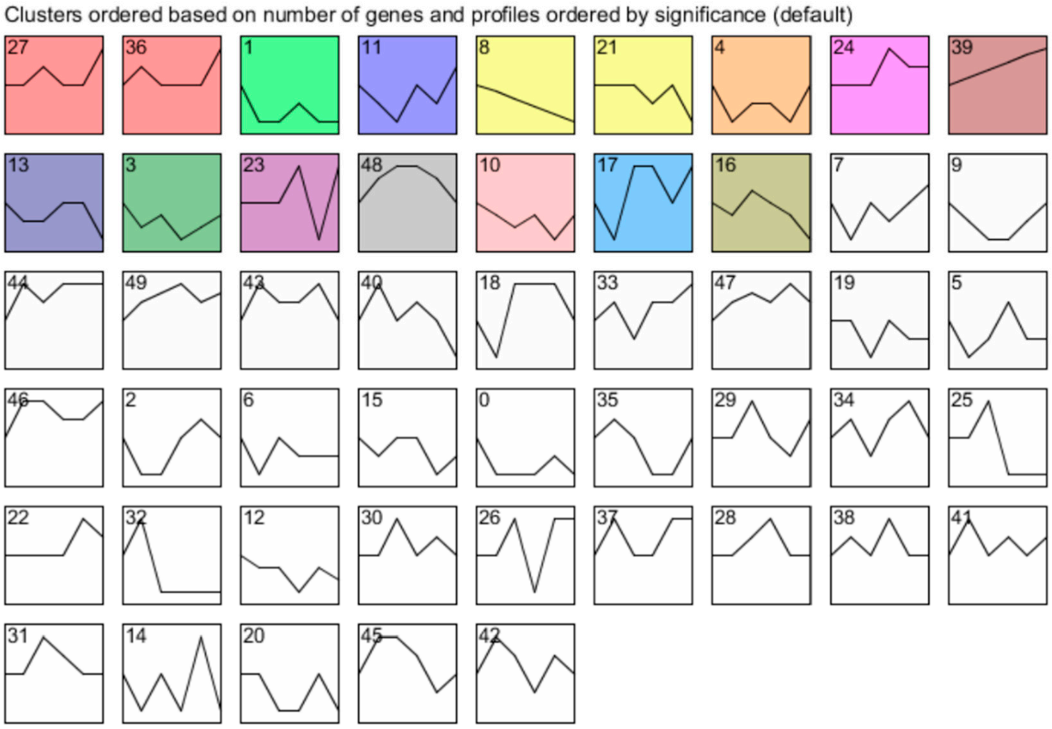

Figure S4. Trend analysis of 27174 genes of six jujube fruit varieties ('bianshizao', 'jingcangyihao', 'xinmopan', 'zidantou', beijingdamaya', and 'zhenhulu') at the D40 developmental stage.

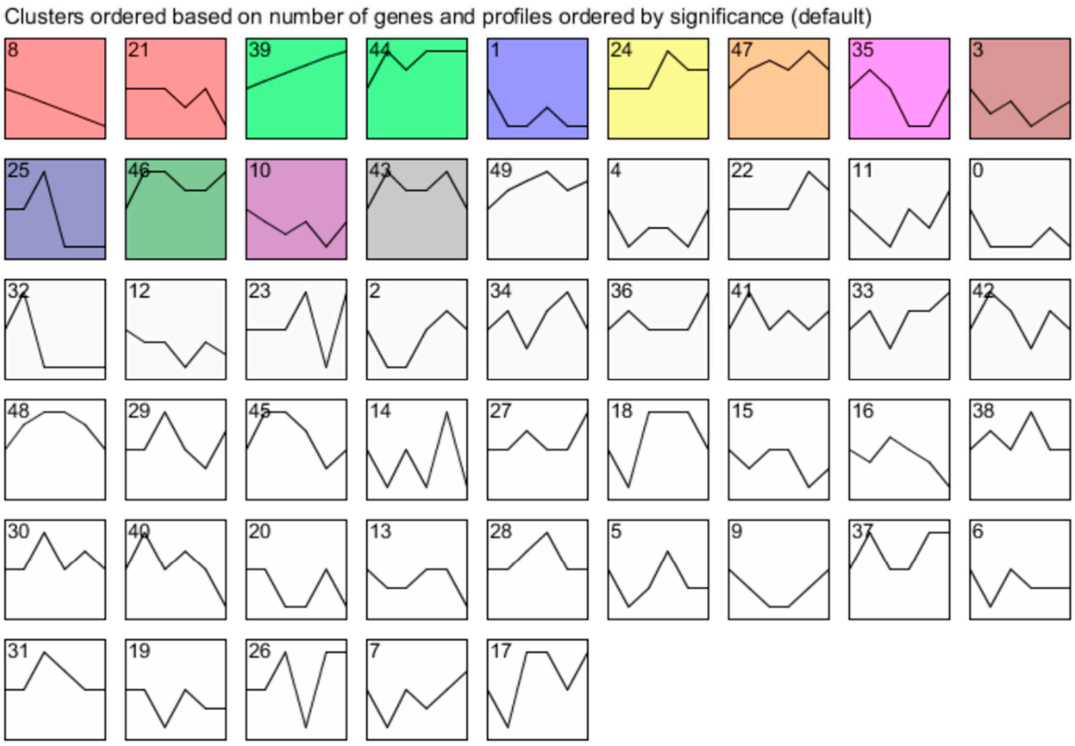

Figure S5. Trend analysis of 27174 genes of six jujube fruit varieties ('bianshizao', 'jingcangyihao', 'xinmopan', 'zidantou', beijingdamaya', and 'zhenhulu') at the D55 developmental stage.

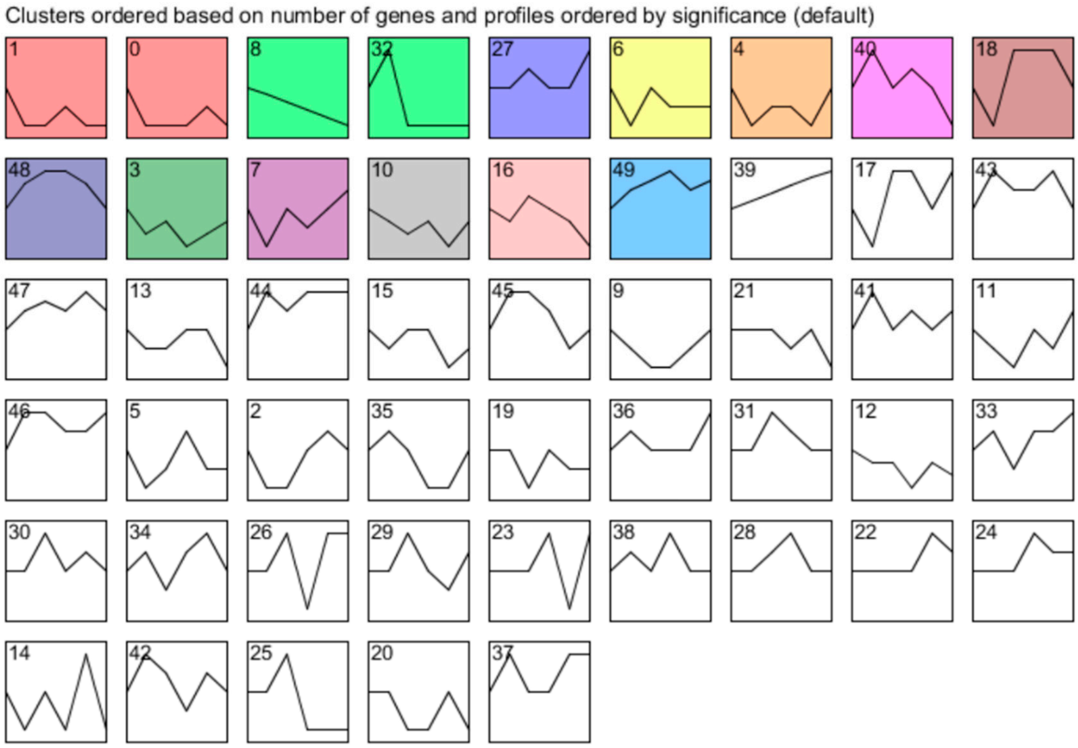

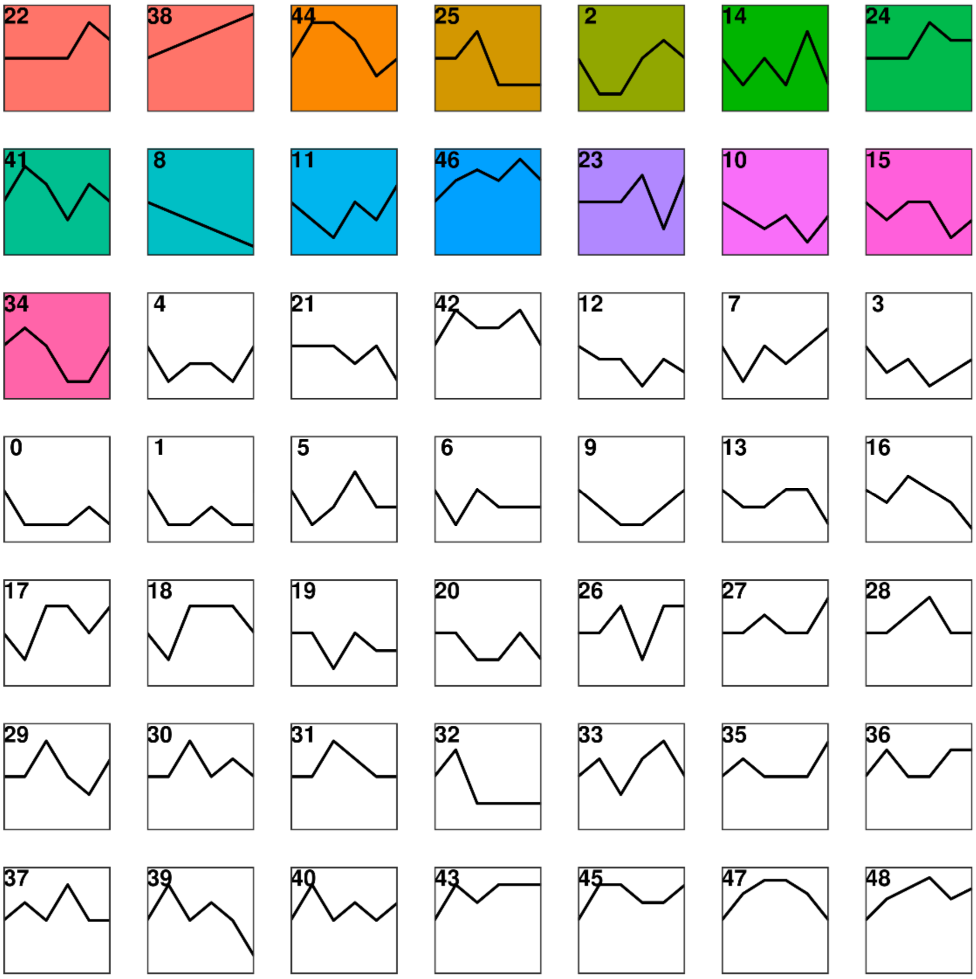

Figure S6. Trend analysis of 27174 genes of six jujube fruit varieties ('bianshizao', 'jingcangyihao', 'xinmopan', 'zidantou', beijingdamaya', and 'zhenhulu') at the D70 developmental stage.
